# Preconceptional Iron Intake and Gestational Diabetes Mellitus

**DOI:** 10.3390/ijerph13060525

**Published:** 2016-05-24

**Authors:** Anne Marie Darling, Allen A. Mitchell, Martha M. Werler

**Affiliations:** 1 Department of Epidemiology, Boston University School of Public Health, Boston, MA 02118, USA; amdarlin@bu.edu; 2 Slone Epidemiology Center at Boston University, Boston, MA 02215, USA; allenmit@bu.edu

**Keywords:** iron, gestational diabetes, diet, pregnancy

## Abstract

Our objective was to assess the impact of preconceptional heme and non-heme iron on gestational diabetes mellitius (GDM) in the Boston University Slone Epidemiology Birth Defects Study (BDS). This retrospective cohort analysis included 7229 participants enrolled in the BDS between 1998 and 2008 who gave birth to non-malformed infants and were free of pre-existing diabetes. All data were collected through structured interviews conducted within 6 months of delivery. Calorie-adjusted and multivariable odds ratios (ORs) and 95% confidence intervals (CIs) were calculated using logistic regression models. Preconceptional dietary heme iron was modestly associated with an elevated risk of GDM among those (multivariable OR comparing the fifth quintile to the first: 1.55; 95% CI 0.98, 2.46). Conversely, preconceptional dietary non-heme iron was associated with a decreased risk of GDM among those in the fifth quintile of intake compared to the first (multivariable OR: 0.48; 95% CI 0.28, 0.81). Women who consumed supplemental iron during preconception also had a decreased risk of GDM (multivariable OR: 0.78; 95% CI 0.60, 1.02). In conclusion, our data support a positive association between preconceptional heme iron intake and GDM and an inverse association between preconceptional non-heme iron intake from foods and preconceptional intake from supplements.

## 1. Introduction

Iron is an essential nutrient for a healthy pregnancy, but research suggests that excess iron may adversely affect maternal and fetal health [1,2], including the development of gestational diabetes mellitius (GDM). A positive association between ferritin, a marker of body iron stores, and GDM has been reported in several small case-control and prospective studies [3,4,5,6,7,8].

Determinants of body iron stores include physiological iron demands, dietary iron supply, and the ability of gut mucosal cells to regulate iron absorption [9]. Among these factors, only dietary iron supply is potentially modifiable. Given the substantial short-term and long-term risks to both the mother and fetus associated with gestational diabetes, it is important to understand the extent to which maternal iron intake from foods and supplements may increase the risk of GDM [10,11].

In studies of iron intake and GDM, the bioavailability of ingested iron may be more important to consider than the total amount consumed. Although heme iron, which is obtained from animal sources, represents only 10%–15% of dietary iron in meat-eating populations, it may account for nearly one third of absorbed iron [12]. Heme iron is more readily absorbed because it is bound within the heme porphyrin ring; this prevents other molecules from binding to it and reducing its bioavailability. Because non-heme iron, found in cereals, vegetables, fruits, grains, and dairy products, has no such protection, molecules present in other meal components can bind to it and render it relatively insoluble [9]. As a result, heme iron intake is positively related to serum ferritin levels, whereas total iron and non-heme iron are not [13].

Only two studies have separately examined the relationship between long-term or periconceptional heme and non-heme iron intake and GDM [14,15]. In both, only heme iron was associated with an increased risk of GDM. Another study reported an increased risk of GDM associated with total iron intake but unlike those previously mentioned, this study did not fully control for other dietary factors that could explain the association [16]. For example, foods high in heme iron such as red and processed meats also contain saturated fat and cholesterol, both of which may have deleterious effects on insulin sensitivity and β cell function [17] and should therefore be considered as potential confounders in analyses of heme iron and GDM. The results from studies of the relationship between intake of iron supplements, which generally contain non-heme iron in the form of ferrous sulfate or ferrous fumarate, and GDM have been inconsistent [15,18,19,20,21], though the two randomized trials to examine this relationship have found no evidence of an association.

We used data from the Slone Epidemiology Center Birth Defects Study to examine whether preconceptional heme iron or non-heme iron, or iron intake from supplements, was associated with the risk of GDM. We further explored whether there was evidence of a joint effect of iron intake and factors related to glucose tolerance, oxidative stress, and iron absorption on GDM risk. These factors included BMI, smoking, vitamin C (which enhances iron absorption), and calcium (which inhibits it).

## 2. Materials and Methods

We performed a retrospective cohort study of mothers of non-malformed infants who were enrolled as controls between 1998 and 2008 in the Slone Epidemiology Center Birth Defects Study, an on-going case-control study in the United States and Canada [22,23,24]. The study obtained IRB approval from Boston University School of Public Health. Institutional review board approval was obtained from each of the participating institutions. Controls were randomly selected each month from study hospitals’ discharge lists or from statewide birth records. We excluded women who had multiple gestations (*n* = 192), had pre-existing diabetes (*n* = 42), whose food frequency questionnaires indicated unrealistic values for caloric intake (<500 or >4000 kcal/day, *n* = 613), or who had incomplete information on total dietary iron intake and other key covariates (*n* = 328). The final analytic cohort comprised 7229 participants. 

### 2.1. Exposure Assessment

Trained study nurses conducted computer-assisted telephone interviews with participants within 6 months of delivery. The interviews ascertained information on socio-demographic factors, illnesses during pregnancy, details of prescription and over-the-counter medications used (including vitamins), reproductive history, behavioral risk factors, and diet. Dietary data for the 6-month period prior to pregnancy were collected using a modified 58-item Willett food frequency questionnaire (FFQ), administered by the interviewers, that assessed the consumption frequency and portion size of each item. The Willett FFQ is a validated and commonly-used dietary questionnaire in epidemiologic studies [25]. Nutrient values for individual foods were obtained from the Harvard University Food Composition database. Average daily dietary intake of heme iron and total iron were summed for each participant based on her reported frequency of eating each food. Average daily dietary intake of non-heme iron was calculated for each participant by subtracting her average daily heme iron intake from her average total iron intake. We categorized participants into quintiles of heme and non-heme iron intake based on the intake distribution of women who did not develop GDM. Those who reported any supplemental intake of iron either alone or as part of a multivitamin supplement during the 4 weeks prior to their last menstrual period were considered preconceptional iron supplement users.

### 2.2. Outcome and Covariate Assessment

GDM was defined as the reported onset of diabetes mellitus during the index pregnancy. Socio-demographic and behavioral factors considered in this analysis included race/ethnicity (non-Hispanic white, non-Hispanic black, Hispanic, other), education (less than high school, high school, more than high school), maternal age (<20, 20–29, 30–39, or 40–49 years), family income in 2005 U.S. dollars ($15,000, $15,000–$29,999, $30,000–$44,999, or ≥$45,000, unknown, or refused), smoking during pregnancy (yes, no, quit during pregnancy), alcohol use during pregnancy (yes, no, quit before 24 weeks of pregnancy ), and body mass index. Body mass index (BMI) was calculated based on the mother’s self-reported height and pre-pregnancy weight. Preconceptional dietary covariates included quintiles of saturated fat, polyunsaturated fat, dietary fiber, cholesterol, vitamin C, glycemic index, glycemic load, and daily servings of red/processed meat.

### 2.3. Statistical Analysis

Logistic regression models were used to calculate odds ratios (ORs) and 95% confidence intervals (CIs) for the relationship between iron intake and GDM in the upper quintiles of intake compared to the lowest quintile and for supplement users compared to non-users. We first ran models adjusted for calorie intake only. Both forms of preconceptional dietary iron, iron supplementation, total energy intake, and any covariates described above that changed the estimated association by at least 10% were included in the final multivariable model. Only BMI and cholesterol intake, intake met the criterion as potential confounders of the association between heme iron and GDM. Maternal age, BMI, fiber intake, glycemic index met the criterion for confounders in the non-heme iron association with GDM. Maternal age, race and income met the criterion for confounders in the supplemental iron association with GDM. We conducted tests for linear trends in the associations between GDM and quintiles of heme and non-heme iron by modeling the median values of each intake category as continuous variables and obtaining *p*-values from Wald’s test.

We also evaluated the continuous relationship between GDM and the average daily intakes (milligrams per day) of heme and non-heme iron. To do so, we first examined the possibility of a non-linear relationship between heme iron and non-heme iron intake in a restricted cubic spline analysis. We used a likelihood ratio test to compare an adjusted logistic regression model with only linear terms for iron intake to one with both linear and cubic spline terms for iron intake. These tests failed to reject the null hypothesis that an association with GDM, if any, was linear for both heme and non-heme iron. Therefore, we modeled continuous dietary intake of both heme and non-heme iron using only linear terms. We calculated crude and multivariable ORs and 95% confidence intervals per 1 mg/day increase in intake. Continuous multivariable models were adjusted for the same covariates as the categorical models.

We also assessed the possibility that BMI (<25 = referent *vs.* ≥25), smoking (never = referent *vs.* ever), dietary vitamin C intake (below median = referent *vs.* median or above) and dietary calcium intake (below median = referent *vs.* median or above) may have synergistic or antagonistic effects on the association between dietary iron intake and GDM. In each of these analyses, we categorized subjects into 10 unique groups based on their quintile of iron intake and exposure level of the second variable of interest. We considered those who were in the lowest quintile of iron intake and in the reference group for the second exposure of interest as the reference group for this analysis and calculated ORs for GDM relative to each of the other 9 categories. We then calculated the relative excess risk due to interdependence (RERI) and 95% CIs using adjusted odds ratios obtained from logistic regression.

Because GDM is usually diagnosed after 12 weeks of pregnancy, we lastly examined the sensitivity of our findings to the timing of GDM onset. In this analysis, we limited our case definition to women with a reported GDM onset date that was at least 84 days following the date of their last menstrual period.

## 3. Results

Table 1 shows the distribution of socio-demographic, behavioral, and dietary covariates according to quintiles 1, 3, and 5 of preconceptional heme and non-heme iron intake. Women who consumed the highest amount of heme iron had higher BMIs on average and were more likely to be African American, have an annual income of less than $15,000, and have completed 12 or fewer years of education than women who consumed the least amount of heme iron. Those who consumed the highest amounts of non-heme iron had lower BMIs on average and were more likely to be Hispanic than those who consumed the lowest amounts of non-heme iron. Although those in the highest quintiles for both heme and non-heme iron intake had higher average glycemic loads, and consumed more of each dietary factor on average than those in the lower quintiles, those in the highest quintile of heme iron consumed more saturated fat, cholesterol, and red/processed meat on average compared to those in the highest quintile of non-heme iron, whereas those in the highest quintile of non-heme iron intake consumed more polyunsaturated fat, dietary fiber, vitamin C, and calcium on average than those in the lower quintiles. The mean (SD) time between delivery and the study interview was 4.86 (0.71) months. A total of 316 women (4.4%) reported GDM.

In calorie-adjusted models, the odds of GDM increased with increasing quintiles of preconceptional heme iron intake (*p*-trend = 0.02) (Table 2). The odds of GDM were increased by more than twofold in the highest quintile compared to the lowest (OR 2.51, 95% CI 1.67, 3.76). When we adjusted for BMI, cholesterol intake, and red/processed meat intake, the magnitude of this association was attenuated but the risk remained elevated (multivariable OR 1.55, 95% CI 0.98, 2.46). The continuous model showed that each mg/day increase in dietary heme iron was associated with 1.41 times the odds of GDM (95% CI 1.05, 1.89).

By contrast, preconceptional non-heme iron intake was associated with a reduced risk of GDM. In the final multivariable model, with the lowest quintile as reference, adjusted ORs for GDM across increasing quintiles of non-heme iron intake were 0.73 (95% CI 0.50, 1.07), 0.55 (95% CI 0.35, 0.86), 0.65 (95% CI 0.40, 1.02), and 0.48 (95% CI 0.28, 0.81). The continuous model showed that each mg per day increase in non-heme iron intake was associated with a slight decrease in the odds of GDM (multivariable OR 0.95, 95% CI 0.92, 0.99). Similarly, those who used supplemental iron during the preconceptional period had 0.78 times the odds of GDM (95% CI 0.60, 1.02) as non-users.

Having a BMI of greater than 25 was associated with 2.47 times the odds of developing GDM (95% CI 1.96, 3.10). Relative to women in the lowest quintile of preconceptional heme iron consumption who had a BMI of less than 25, the joint impact of heme iron consumption and a BMI of 25 or greater on the odds of GDM was 3.68-fold (Table 3). Although the magnitude of the odds ratio for the doubly-exposed women was larger than those for either risk factor alone, there was little evidence of a synergistic effect (RERI = 0.59, 95% CI −1.10, 2.28). When compared to participants with a BMI less than 25 and non-heme iron intake in the lowest quintile, participants with a BMI of less than 25 in all other quintiles (Q2–Q5) had a reduced risk of GDM that was not observed for women with a BMI of 25 or greater at any level of non-heme iron intake (RERI = −0.35, 95% CI −1.22, 0.52). Neither the associations between GDM and either heme iron nor non-heme iron appeared to differ according to smoking status or consumption of dietary calcium or vitamin C (data not shown). 

When we restricted the case definition to women who reported the onset of GDM after 12 weeks of pregnancy (*n* = 291), the association between preconceptional heme iron intake and GDM became stronger and fully consistent with an adverse impact of heme iron on GDM development (multivariable OR, quintile 5 *vs.* quintile 1: 1.87 (95% CI 1.15, 3.03)). With the same restricted case definition, the results for dietary non-heme and supplemental iron intake were similar to those seen when all reported GDM diagnoses during pregnancy were included. 

## 4. Discussion

We observed at least a 50% reduction in the risk of GDM among women who consumed the highest amounts of dietary non-heme iron compared to those who consumed the lowest level during the preconception period. Intake of supplemental iron, which is most commonly derived from plant sources, was also associated with a modest decrease in the risk of GDM. Our data do not suggest the presence of a synergistic or antagonistic relationship with either type of iron and BMI, smoking, dietary calcium intake, or dietary vitamin C intake. Only two previous studies have examined the separate contribution of heme and non-heme iron to GDM. Qiu *et al.* [14] examined the relation between periconceptional and early pregnancy dietary heme and non-heme iron and GDM in 3148 participants in a prospective cohort of pregnant women attending prenatal care clinics in Washington State. They found an over threefold increase in risk of GDM among women reporting the highest heme iron intake compared to those reporting the lowest. Similarly, Bowers *et al.* [15] found an increased risk of GDM with increasing quintiles of long-term heme iron intake among 13,475 participants in the Nurses’ Health Study II cohort. In both studies, the risk of GDM remained elevated after adjustment for red meat and its constituents, such as saturated fat and cholesterol. Our results appear to agree with these findings, even though the width of the confidence intervals in our study prevents us from ruling out a null or protective association among non-obese women (the precision of our results was limited by a lower frequency of GDM in our study). In addition, some participants who had undetected preexisting diabetes may have been misclassified as having GDM. This misclassification likely would have occurred irrespective of iron status, so it would likely have biased our results toward the null. Further, when we limited our case definition to diagnoses of GDM after 12 weeks of pregnancy, we observed a stronger association with a 95% confidence interval that excluded the null.

The relationship between non-heme iron and GDM differed in the two prior studies. Consistent with our results, Qiu *et al.* [14] reported that non-heme iron intake appeared to be inversely associated with GDM, though Bowers *et al.* [15] observed no multivariable association between non-heme iron and GDM risk.

A positive association between heme iron and GDM is biologically plausible, though the mechanisms underlying this association are not fully understood. Iron may decrease insulin extraction and metabolism in the liver, which could lead to hyperinsulinemia [26]. Iron may also induce insulin deficiency through a pro-oxidant effect on pancreatic islet cells [27,28]. In light of this proposed oxidative pathway, we examined the possibility that the antioxidant vitamin C modified the association between iron and GDM, but we found that dietary intake of vitamin C above the median had no measurable effect on GDM. While the joint association of obesity and high heme iron intake was larger than that of either obesity or high heme iron intake alone, the small magnitude of the RERI suggested that no that biologic synergism exists between the two risk factors. Another possible explanation for our findings is that high heme iron intake may be indicative of an overall dietary pattern that increases the risk of GDM.

Potential mechanisms for an inverse association between non-heme iron and GDM, an observation which is inconsistent with the results of randomized trials [20,21], are unknown. In our study, non-heme iron intake may be serving as a proxy for other dietary components found foods rich in non-heme iron. Polyunsaturated fat [29] and fiber [30] have previously been associated with a reduced risk of GDM. However, the inclusion of these variables in the multivariate model had little impact on our results, and neither was independently associated with GDM.

Non-heme iron intake may also serve as a proxy for a dietary pattern that prevents GDM. In the Nurses’ Health Study II, the “prudent” diet containing higher amounts of vegetables, legumes, fruits and poultry) was associated with a lower risk of GDM compared to a “Westernized” diet heavy in red and processed meat [31]. In another study, a higher score on a Mediterranean diet index, indicating greater consumption of vegetables, whole grains, beans, nuts and seeds, and healthy fats, was associated with a reduced incidence of GDM [32]. Those who follow “prudent” or “Mediterranean” dietary patterns likely consume substantial amounts of non-heme iron, though non-heme iron itself may not exert a protective influence on GDM development.

We also cannot rule out the possibility that this association remains confounded by factors related to a healthy lifestyle for which we were unable to adjust. For example, we had no information on participants’ physical activity levels. Bowers *et al.* [15], who observed an age-adjusted but not multivariable association between non-heme iron and GDM, included physical activity in their adjusted models. At the same time, the inclusion of other lifestyle related covariates in our models, such as BMI, alcohol consumption during pregnancy, and smoking during pregnancy did not materially change the association.

Our study is also limited by measurement error since both diet and GDM were collected by retrospective self-reporting. Our results could be biased away from the null if those who had GDM over-reported meat consumption and under-reported the consumption of foods high in non-heme iron. We believe this is unlikely, however, since participants were unaware of any specific hypotheses related to iron and GDM at the time of the interview. Our results are more likely to have been affected by non-differential errors in recall due to the time elapsed between the preconceptional period and the interview. We would expect that any such errors would bias our results toward the null. While no validation studies have thus far been undertaken on self-reported GDM in this cohort, we believe that this variable was reported with reasonable accuracy. Screening for gestational diabetes is performed as part of standard pre-natal care through the administration of an oral glucose tolerance test between 24 and 28 weeks of pregnancy, and it is likely that self-reported GDM refers to a diagnosis received based on the results of this test. This test has an established blood glucose cutoff of 140 mg/dL, so we would not expect that the diagnostic practices for GDM would vary among hospitals.

Another limitation arises from the measurement of pre-pregnancy diet. Women often change their diet during pregnancy, which may be the more relevant time frame for iron to affect the development of GDM. Unfortunately, data were not available on changes in dietary intakes during pregnancy. Further limitations include residual confounding by dietary and lifestyle factors and our inability to account for frequency, dose, or duration of iron supplementation. Strengths of our study include its large sample size of participants randomly selected from the study hospitals’ discharge lists and statewide birth records that is likely representative of pregnant women in the study areas, detailed collection of dietary data, and use of a validated dietary assessment tool. 

## 5. Conclusions

Our findings lend some support to previous research suggesting that heme iron is associated with an increased risk of GDM. Our observed reduction in risk of GDM associated with non-heme iron requires confirmation in other studies, and if that occurs, research is needed to determine whether this association may be explained by other factors associated with diets high in non-heme iron.

## Figures and Tables

**Table 1:** Baseline characteristics by quintiles **^a^** 1, 3, and 5 of total preconceptional dietary iron intake (*n* = 7229).

Variable ^b^	Heme Iron Intake			Non-Heme Iron Intake		
	Q1	Q3	Q5	Q1	Q3	Q5
Age (years)	28.88 (6.07)	29.51 (5.62)	28.52 (5.84)	28.58 (6.22)	29.41 (5.66)	29.60 (5.67)
BMI (kg/m^2^)	22.93 (5.90)	23.77 (6.17)	24.45 (6.71)	24.13 (6.50)	23.71 (601)	23.47 (5.92)
Race						
White	1074 (72.76)	1138 (75.36)	867 (59.22)	1068 (72.75)	1036 (72.25)	972 (67.83)
African American	96 (6.50)	103 (6.82)	159 (10.86)	122 (8.31)	110 (7.67)	110 (7.68)
Hispanic	170 (11.52)	182 (12.05)	291 (19.88)	175 (11.92)	190 (13.25)	238 (16.61)
Other	136 (9.21)	87 (5.76)	147 (10.04)	103 (7.02)	98 (6.83)	113 (7.89)
Years of education						
<12	142 (9.62)	101 (6.69)	146 (9.97)	138 (9.40)	100 (6.97)	111 (7.75)
12	246 (16.67)	247 (16.36)	322 (21.99)	324 (22.07)	232 (16.18)	225 (15.70)
≥12	1088 (73.71)	1162 (76.95)	996 (68.03)	1006 (68.53)	1102 (76.85)	1097 (76.55)
Annual income						
<$15,000	117 (7.93)	91 (6.03)	159 (10.86)	128 (8.72)	101 (7.04)	102 (7.12)
$15,000 to $24,000	51 (3.46)	43 (2.85)	60 (4.10)	61 (4.16)	47 (3.28)	52 (3.63)
$25,000 to $34,000	59 (4.00)	66 (4.37)	92 (6.28)	58 (3.95)	55 (3.84)	91 (6.35)
≥$35,000	983 (66.60)	1122 (74.30)	896 (61.20)	961 (65.46)	1033 (72.04)	983 (68.60)
Unknown	244 (16.53)	170 (11.26)	235 (16.05)	237 (16.14)	175 (12.20)	187 (13.05)
Refused	22 (1.49)	18 (1.19)	22 (1.50)	23 (1.57)	23 (1.60)	18 (1.26)
Alcohol use during pregnancy (%)						
No	709 (47.97)	635 (42.05)	729 (49.80)	663 (45.16)	642 (44.77)	656 (45.78)
Yes	726 (49.19)	830 (54.97)	702 (47.95)	772 (52.59)	743 (51.81)	738 (51.50)
Quit before 24 weeks	42 (2.85)	45 (2.98)	33 (2.25)	33 (2.25)	49 (3.42)	39 (2.72)
Smoking during pregnancy						
No	1017 (68.90	992 (65.70)	961 (65.64)	906 (61.72)	958 (66.81)	984 (68.67)
Yes	107 (7.25)	101 (6.69)	151 (10.31)	136 (9.26)	111 (7.74)	95 (6.63)
Quit before 24 weeks	352 (23.85)	417 (27.62)	352 (24.04)	426 (29.02)	365 (25.45)	354 (24.70)
Diet						
Total iron (mg/day)	10.13 (5.38)	11.75 (4.80)	14.88 (5.24)	6.08 (1.34)	11.25 (0.80)	20.29 (4.58)
Heme iron (mg/day)	0.28 (0.14)	0.73 (0.06)	1.50 (0.44)	0.58 (0.30)	0.81 (0.42)	0.98 (0.59)
Nonheme iron (mg/day)	9.85 (5.39)	11.02 (4.80)	13.39 (5.11)	5.50 (1.22)	10.45 (0.67)	19.32 (4.56)
Total calories (kcal/day)	1250.84 (451.09)	1500.84 (439.82)	1979.10 (569.48)	1058.08 (312.75)	1570.00 (398.33)	1996.74 (590.33)
Saturated fat (g/day)	14.14 (6.66)	18.60 (6.73)	26.33 (8.45)	13.68 (5.58)	19.79 (7.13)	24.16 (9.83)
Polyunsaturated fat (g/day)	6.44 (3.37)	7.69 (2.95)	9.81 (3.62)	5.23 (2.06)	8.00 (2.85)	10.21 (4.08)
Dietary fiber (g/day)	15.44 (7.88)	15.99 (6.76)	19.41 (8.02)	9.68 (3.56)	16.66 (5.02)	23.66 (8.41)
Cholesterol (mg/day)	141.27 (84.00)	219.83 (91.97)	337.19 (135.33)	161.42 (152.09)	230.34 (106.73)	280.42 (152.09)
Vitamin C (mg/day)	114.38 (70.71)	124.83 (68.07)	148.05 (82.26)	79.12 (48.16)	127.53 (60.85)	176.53 (87.01)
Calcium (mg/day)	715.83 (365.9)	784.77 (362.83)	875.93 (397.55)	522.38 (268.10)	800.68 (322.86)	1057.73 (405.75)
Red/processed meat (servings/day)	0.17 (0.18)	0.31 (0.27)	0.63 (0.48)	0.25 (0.23)	0.37 (0.35)	0.41 (0.43)
Dietary glycemic index	53.46 (4.58)	53.75 (4.42)	54.33 (4.33)	53.64 (5.23)	53.82 (4.15)	54.09 (3.80)
Dietary glycemic load	98.68 (44.32)	107.84 (46.01)	134.07 (56.67)	73.92 (34.96)	112.55 (39.95)	147.47 (52.61)
Iron supplements	542 (37.31)	548 (36.48)	447 (30.68)	479 (32.81)	553 (38.67)	561 (39.40)

**^a^** Quintile cutoffs were derived from the 6913 non-diabetic pregnancies; **^b^** Continuous variables expressed as mean (SD), categorical variables expressed as *n* (%).

**Table 2:** Calorie-adjusted and multivariate ORs for GDM (*n* = 316) by preconceptional dietary, heme and non-heme iron intake (*n* = 7229).

Type of Iron	Quintile of Intake					
	Q1	Q2	Q3	Q4	Q5	*p*-Trend
**Heme Iron**						
Number of cases	48	52	69	59	88	
Calorie-adjusted OR (95% CI)	1.00 (Ref.)	1.21 (0.81, 1.80)	1.58 (1.08, 2.31)	1.55 (1.04, 2.31)	2.53 (1.70, 3.78)	0.02
Multivariate model 1 **^a^** OR (95% CI)	1.00 (Ref.)	1.08 (0.71, 1.64)	1.28 (0.84, 1.96)	1.18 (0.75, 1.85)	1.55 (0.98, 2.46)	0.49
**Non-Heme Iron**						
Number of cases	83	70	53	60	50	
Calorie-adjusted OR (95% CI)	1.00 (Ref.)	0.81 (0.58, 1.13)	0.60 (0.41, 0.87)	0.66 (0.54, 0.98)	0.53 (0.34, 0.83)	0.13
Multivariate model 2 **^b^** OR (95% CI)	1.00 (Ref.)	0.73 (0.50, 1.07)	0.55 (0.35, 0.86)	0.65 (0.40, 1.02)	0.48 (0.28, 0.81)	0.23

**^a^** Adjusted for total calories (continuous), quintiles of non-heme iron, use of iron supplements, body mass index (<18.5, 18.5–24.9, 25–29.9, ≥30), and quintiles of cholesterol; **^b^** Adjusted for total calories (continuous), quintiles of heme iron, use of iron supplements, age (<20, 20–24, 24–29, 30–34, >34), quintiles of fiber intake and quintiles of glycemic index.

**Table 3:** Multivariate ORs for the joint association between iron intake, body mass index, and GDM (*n* = 7229, cases = 316).

BMI	Heme Iron Quintile	No. of Cases	Multivariate OR ^a^ (95% CI)	Non-Heme Iron Quintile	No. of Cases	Multivariate OR ^b^ (95% CI)
<25	Q1	25	1.00 (Ref.)	Q1	41	1.00 (Ref.)
	Q2	23	1.01 (0.56, 1.81)	Q2	27	0.52 (0.31, 0.89)
	Q3	33	1.29 (0.74, 2.28)	Q3	28	0.53 (0.31, 0.92)
	Q4	24	1.08 (0.59, 2.00)	Q4	23	0.43 (0.23, 0.78)
	Q5	38	1.68 (0.93, 3.01)	Q5	24	0.41 (0.22, 0.76)
≥25	Q1	23	2.41 (1.35, 4.30)	Q1	42	1.57 (0.82, 3.02)
	Q2	29	2.76 (1.57, 4.84)	Q2	43	1.52 (0.77, 3.01)
	Q3	36	3.12 (1.78, 5.46)	Q3	25	0.89 (0.42, 1.88)
	Q4	35	3.03 (1.71, 5.38)	Q4	37	1.34 (0.65, 1.79)
	Q5	50	3.68 (2.08, 6.49)	Q5	26	0.81 (0.37, 1.80)

**^a^** Adjusted for total calories (continuous), quintiles of non-heme iron, use of iron supplements, and quintiles of cholesterol; **^b^** Adjusted for total calories (continuous), quintiles of heme iron, use of iron supplements, age (<20, 20–24, 24–29, 30–34, >34), quintiles of fiber intake and quintiles of glycemic index.

## Abbreviations

The following abbreviations are used in this manuscript:

GDM	Gestational Diabetes Mellitus
FFQ	Food Frequency Questionnaire
BMI	Body Mass Index
CI	Confidence Interval
RERI	Relative Excess Risk due to Interdependence
